# Safety and Efficacy of High Power (> 100W) Microwave Ablation Using the EMPRINT™ HP System: Final Results of a Post-Market Clinical Follow-Up Study

**DOI:** 10.1007/s00270-025-04228-y

**Published:** 2025-10-15

**Authors:** Susan van der Lei, Stella van der Wal, Timothy Neuss, Alessandro Serafini, Ludovico Ambrosi, Hannah H. Schulz, Danielle J. W. Vos, Madelon Dijkstra, Florentine E. F. Timmer, Robbert S. Puijk, Martijn R. Meijerink

**Affiliations:** 1 Department of Radiology and Nuclear Medicine, Amsterdam UMC Location VUmc, De Boelelaan 1117, 1081 HV Amsterdam, The Netherlands; 2 Cancer Center Amsterdam, Research Program, Amsterdam, The Netherlands; 3 Diagnostic and Interventional Radiology Unit, IRCCS Candiolo Institute, Candiolo, TO Italy; 4 Diagnostic Imaging and Radiotherapy Unit, Department of Clinical, Surgical, Diagnostic, and Pediatric Sciences, University of Pavia, Pavia, Italy; 5 Department of Radiology and Nuclear Medicine, OLVG Hospital, Amsterdam, The Netherlands

**Keywords:** Colorectal cancer (CRC), Colorectal liver metastases (CRLM), Hepatocellular carcinoma (HCC), Liver metastases, Thermal ablation, Microwave ablation (MWA)

## Abstract

**Purpose:**

Microwave ablation (MWA) with higher-power settings (> 100 W) aims to create larger, more uniform ablation zones, potentially reducing local tumour progression (LTP). This post-market clinical follow-up study evaluated the safety and efficacy of higher-power settings for MWA of liver tumours.

**Methods:**

Patients with colorectal liver metastases (CRLM) and hepatocellular carcinoma (HCC) treated with MWA (> 100 W) using the EMPRINT^TM^HP generator (Medtronic, Minneapolis, MN, USA) were included. The primary outcome measure was safety, assessed by CTCAE. Higher-power settings were considered safe if complication rates did not exceed those of a historical cohort treated with conventional settings (EMPRINT™; ≤ 100 W). Secondary outcome measures included LTP-free survival (LTPFS), local control (LC), sphericity index (SI) and length of hospital-stay.

**Results:**

One-hundred-and-twenty-three patients were included. Following 97 procedures for CRLM (82 patients), adverse events (AEs) occurred in 13.4%, with serious AEs (SAEs) in 4.1%. In 44 procedures for HCC (41 patients), AEs occurred in 11.4%, with SAEs in 6.8%. The total SAE rate (5.0%) was lower than the historical cohort (5.8%). For CRLM and HCC, 1-, 2-, and 3-year LTPFS was 94.9%, 93.7%, 93.7%, and 95.1%, 92.5%, 92.5%, respectively. LTP occurred in 4.8% of CRLM (vs. 6.9% in historical cohort; *p* = 0.38) and in 6.3% of HCC. LC was 100%. Median hospital-stay was 1 day. Median SI was 0.79 for both CRLM and HCC.

**Discussion:**

This study indicates that higher-power settings (> 100 W) during MWA for liver tumours does not compromise safety or sphericity of the ablation zone. This was supported by low LTP rates and 100% local control.

**Level of Evidence:**

Level 3

**Graphical Abstract:**

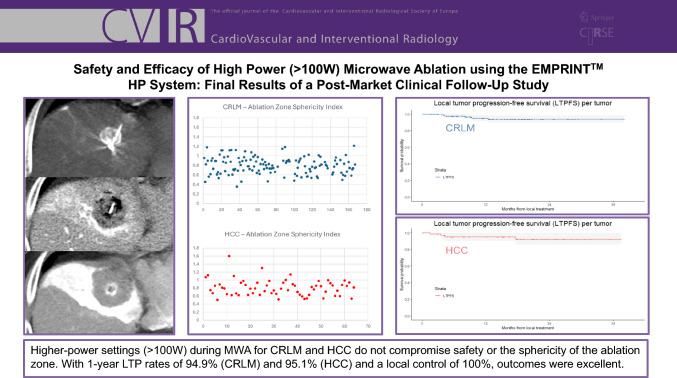

## Introduction

Thermal ablation is a well-established treatment for both primary and secondary liver tumours. Several studies, including the COLLISION trial, have shown that thermal ablation for small-size colorectal liver metastases (CRLM) provides comparable local control while being associated with lower rates of morbidity and mortality compared to surgical resection. For patients with small-size CRLM, thermal ablation is now considered an equally effective first-line treatment as surgical resection [1–7].

Similarly, for hepatocellular carcinoma (HCC), thermal ablation has long been established as a curative-intent treatment for (very) early-stage liver cancer. Its favourable safety profile and high efficacy in eradicating small tumours have led to widespread endorsement in international guidelines [8].

MWA has gained popularity worldwide over RFA due to its superior performance in achieving high temperature heating and reduced heat sink concerns [6, 9]. During RFA, the zone of active tissue heating is confined to a few millimetres around the active electrode, with the remainder of the ablation zone being heated via thermal conduction [10]. In contrast, MWA offers a broader field of power density (up to 2 cm surrounding the probe), resulting in a larger zone of active heating. This expanded zone allows for a more homogeneous zone of tumour cell death, both within the targeted region as well as adjacent to blood vessels [11]. This feature is thought to make MWA less affected by heat sink. MWA harnesses dielectric hysteresis to produce heat. Tissue destruction occurs when tissues are heated to lethal temperatures through an applied electromagnetic field, typically in the range of 900–2.45 GHz. Polar molecules in tissue such as H_2_O are forced to continuously realign with the oscillating electric field, increasing their kinetic energy and, hence, the temperature of the tissue [12, 13].

Recent advancements in the field of MWA, including higher frequency bands (2.45 GHz) and spatial energy control techniques (providing thermal-, field-, and wavelength-control) appear to create more predictable and spherical ablation zones, irrespective of target location, tissue type, or changes in tissue properties during ablation [14–17]. Nonetheless, without probe repositioning, the size of the ablation zone is still frequently insufficient, especially for larger tumours, resulting in inadequate tumour-free margins to ensure long-term tumour progression free survival.

Though improved over time, local tumour progression (LTP) remains a significant limitation, with efficacy decreasing exponentially as tumour size increases [18, 19]. The use of higher energies may prove superior for both smaller-sized (< 3 cm) liver tumours as well as enable the curative-intent ablation of larger-size liver tumours. The recently introduced high power (HP) EMPRINT^TM^ MWA generator (EMPRINT^TM^HP, Medtronic, Minneapolis, USA), permitting ablations at power-settings exceeding 100 Watt (maximum 150 W), was implemented with the aim of enhancing local disease control.

This post-market clinical follow-up study was designed to confirm improvements in oncological outcomes and assess whether these gains come at the expense of a compromised safety profile or diminished predictability of ablation-zone sphericity for the two most commonly treated disease entities in the liver; HCC and CRLM.

## Materials and Methods

### Study Design

This single-arm safety study included all patients with CRLM and HCC treated with MWA at higher power settings (> 100 W) using a commercially available microwave generator (EMPRINT^TM^HP) (Medtronic, Minneapolis, MN, USA) from two prospectively maintained databases (AmCORE, HepaCARE) at the Amsterdam University Medical Centres, the Netherlands, a tertiary referral medical centre for gastrointestinal and hepatobiliary cancer. Supplementary data were collected from the electronic patient records. Data extraction and data reporting are in accordance with the ‘Strengthening the Reporting of Observational studies in Epidemiology’ (STROBE) guideline [20]. The affiliated Institutional Review Board granted permission for this study (METC 2024.0010).

### Patient Selection and Data Collection

Patients with CRLM were considered eligible for thermal ablation if the CRLM were considered unsuitable for partial hepatectomy and if all metastatic sites could be eradicated by thermal ablation or by thermal ablation plus partial hepatectomy. Extrahepatic disease was allowed as long as the extrahepatic disease was also amenable for curative intent eradication.

Patients with HCC were considered eligible for thermal ablation in case of very early stage disease (solitary HCC < 2 cm), early stage disease with portal hypertension or early stage disease and on the waiting list to be transplanted, according to the Barcelona Center for Liver Cancer staging system (BCLC-A) [8]. All patients were assessed by a multidisciplinary team (MDT) consisting of medical oncologists, radiation oncologists, diagnostic and interventional radiologists and hepatobiliary and/or oncological surgeons.

### Thermal Ablation Procedure and Follow-up

A CT-hepatic arteriography (CTHA) guided percutaneous approach was preferred. All CTHA-guided percutaneous ablations were in accordance with the instructions for use as provided by the manufacturer and the CIRSE quality improvement guidelines [21]. Conformal to the CIRSE standards of practice on thermal ablation of liver tumours, the intended minimum tumour-free ablation margin was > 1 cm and the minimum realized tumour-free ablation margin to claim technical success was 5 mm [21]. Ablation zone margins were calculated with confirmation software using rigid 3D image-registration (Syngo Fusion, Siemens, Erlangen, Germany) directly after the ablation with the aim of performing a complementary overlapping ablation where necessary. Follow-up contrast enhanced (ce) CT, 18F-FDG-PET-CT and/or ceMRI scans were performed every 3–4 months in the first year, every 6 months in the second and third year, and every 12 months in the fourth and fifth year following thermal ablation. Power settings were determined at the discretion of the operator, based on tumour size and morphology, the proximity of critical structures and healthy liver parenchyma.

### Statistical Analysis

It was hypothesized that higher power settings (> 100 W) would be similar in terms of safety (primary outcome measure) compared to a historical cohort of patients treated with the conventional EMPRINT™ generator, in which 5.8% experienced ablation-related serious adverse events (SAEs). Adverse events were reported using the Common Terminology Criteria for Adverse Events (CTCAE) [22]. CTCAE grades 1 or 2 were defined as minor adverse events, whereas CTCAE grades 3 through 5 were defined as serious adverse events. The historical control group was derived from a subgroup of patients with CRLM treated with the conventional generator at 100W in the randomized controlled COLLISION trial [4]. For the higher power generator, a true SAE rate, irrespective of underlying disease, of up to 7.5% was considered acceptable (predefined threshold), based on a slightly higher risk of adverse events (AEs) due to the creation of larger ablation zones (true proportion, *P* = 0.075). The non-inferiority margin (*ɗ*) was set at 0.1. The calculated sample size was 123 patients.

Secondary outcome measures, all assessed per disease entity and for CRLM compared to the historical cohort (control group), included complications, technical success, local tumour progression-free survival (LTPFS), local control (LC), overall survival (OS), distant progression-free survival (DPFS), sphericity index (SI), number of probe repositionings, minimal ablation zone margins, and length of hospital stay. The SI was defined as the ratio between the diameters of three axes; ((SAD1 + SAD2)/2 × LAD), as previously described by Hendriks et al. [23]. An SI of 1 denoted a perfectly spherical ablation, whereas an SI < 1 indicated a more elliptical ablation shape. For CRLM, outcomes were considered non-inferior in terms of efficacy if the rates of technical success, LTPFS, and LC were equal to or better than those reported in the control group.

Continuous variables were summarized using standard statistics, including means, standard deviations, medians, and ranges. Categorical variables were summarized using frequencies. *P* values below 0.05 were considered significant. LTPFS and LC were estimated using the Kaplan–Meier method with corresponding two-sided 95% confidence intervals (CIs) for survival proportions. Complications were graded from 1 to 5 according to CTCAE version 5.0 [22]. SPSS® Version 28.0 (IBM®, Armonk, New York, NY, USA) [24] and R version 4.0.3. (R Foundation, Vienna, Austria) [25] were used to perform statistical analyses.

## Results

From March 2021 until April 2024 a total of 123 patients, identified from the prospective AmCORE and HepaCARE databases, received thermal ablation using higher power settings (> 100 W). A total of 82 patients were treated for CRLM, and 41 for HCC.

Among patients with CRLM, the mean age at treatment was 67.4 years (± 11.4). The cohort consisted of 65.9% (*n* = 54) males and 34.1% females (*n* = 28), with 65.9% having an ASA score of 2. Regarding primary tumour location, 26.8% were right-sided, 41.5% were left-sided, and 30.5% were rectal carcinomas. A total of 82 patients underwent 97 thermal ablation procedures using the higher-power generator for at least 1 CRLM. Of 204 CRLM treated, 168 CRLM were ablated using higher power settings (> 100 W), while 36 tumours were treated with a power setting of 100 W. The median follow-up time of patients alive at last follow-up was 16.8 months (range 1.8–37.0). Patient-, disease- and tumour-related characteristics are reported in Table 1.

**Table 1:** Patient-, disease- and tumour characteristics CRLM

Patient-related characteristics	Patients with CRLM
Age, years (mean, SD)	67.4 (±11.4)
Sex (*N*, %)	–
Male	54 (65.9%)
Female	28 (34.1%)
ASA score (*N*, %)	–
1	1 (1.2%)
2	54 (65.9)
3	27 (32.9)
Charlson’s comorbidity index	–
None	24 (29.3%)
Minor	33 (40.2%)
Major	8 (9.8%)
Missing	17 (20.7%)
BMI (median, range)	25.9 (16.87–42.68)
*Disease-related characteristics*	
Primary tumour location	–
*Right-sided*	22 (26.8%)
*Left-sided*	34 (41.5%)
*Rectum*	25 (30.5%)
*Duodenum*	1 (1.2%)
Synchronous	40 (48.8%)
Metachronous	42 (51.2)

Categorical variables are reported as number of patients (%), continuous variables are reported as mean (SD) median (range), ASA = American society of anaesthesiologists, BMI = Body mass index

The patients with HCC had a mean age of 70.6 years (± 8.1) at the time of treatment. The majority were male (78%, *n* = 32) compared to females (22%, *n* = 9). An ASA score of 3 was most common, and was reported in 82.9% of the patients. A total of 41 patients underwent 44 thermal ablation procedures using the higher-power generator to for at least 1 HCC. Of 73 HCCs, 64 were treated with power settings > 100 W, while 9 tumours were treated at 100 W. The median follow-up time for patients alive was 21.1 months (range 5.8–37.5). Patient-, disease- and tumour-related characteristics are reported in Table 2.

**Table 2:** Patient-, disease- and tumour characteristics HCC

Patient-related characteristics	Patients with HCC (*N*=41)
Age, years (mean, SD)	70.6 (±8.1)
Sex (*N*, %)	–
Male	32 (78%)
Female	9 (22%)
ASA score (*N*, %)	–
1	0
2	6 (14.6%)
3	34 (82.9)
4	1 (2.4)
BMI (mean, SD)	26.3 (±4.2)
*Disease-related characteristics*	
AFP (median, range)	7.0 (2.0-1700)
Extrahepatic metastases	0 (100%)
LI_RADS	–
*4*	7 (17.1%)
*5*	34 (82.9%)
Underlying liver disease	–
*No*	3 (9.8%)
Yes	38 (92.7%)
Alcohol	16 (39.0%)
MASH	11 (26.8%)
Hepatitis B	5 (12.2%)
Hepatitis C	9 (22.0%)
Cirrhosis	37 (90.2%)
Encephalopathy	4 (9.8%)
Ascites	6 (14.6%)
Disease specific WHO	–
0	27 (65.9%)
1	11 (26.8%)
2	3 (7.3%)
Child Pugh Score	–
5	18 (43.9%)
6	16 (39.0%)
7	2 (4.9%)
8	1 (2.4%)
9	0
10	1 (2.4%)
Missing	3 (7.3%)
Meld Score	–
6	5 (12.2%)
7	10 (24.4%)
8	7 (17.1%)
9	2 (4.9%)
10	6 (14.6%)
13	2 (4.9%)
14	2 (4.9%)
16	1 (2.4%)
23	1 (2.4%)
Missing	5 (12.2%)

Categorical variables are reported as number of patients (%), continuous variables are reported as mean (SD) or median (range), ASA = American society of anaesthesiologists, BMI = Body mass index, AFP = alfa-foetoproteïne

### Safety

A total of 7 patients experienced 7 SAEs in 141 procedures (5.0%; 4.1% for CRLM, *n* = 4/97; 6.8% for HCC, *n* = 3/44); compared to 6 patients (5.8%) in the historical cohort (104 procedures), with no statistically significant difference observed (*p* = 0.78). Details regarding the complications are provided in Table 3.

**Table 3:** Adverse events (CTCAE)

Complications *N* (%)	CRLM historical cohort (control) (*N* = 104)	CRLM EMPRINT™ HP (*N* = 97)	HCC EMPRINT™ HP (*N* = 44)
None	87 (83.7%)	84 (86.6%)	39 (88.6%)
Yes	17 (16.3%)	13 (13.4%)	5 (11.4%)
Grade 1–2	11 (10.6%)	9 (9.3%)	2 (4.5%)
Grade 3–5	6 (5.8%)	4 (4.1%)	3 (6.8%)^*^

Values are reported as number of procedures (%),CTCAE = Common Terminology Criteria for Adverse Events, *One patient died as a result of fever, oliguria AKD with hyperkalaemia complicated by COVID19 infection, which ultimately led to death of the patient within 90-days following ablation; the ablation was considered related

Of 82 patients with CRLM, 12 experienced AEs following 13 sessions (13/97, 13.4%). Four of these AEs were classified as SAEs, representing 4.1% (4/97) of the cohort. Two events were grade 4: one patient, in whom all 4 CRLM were treated with > 100 W, developed multiple liver abscesses and an abdominal aortic endoprosthesis infection, requiring intravenous antibiotics and prolonged hospitalization; another patient, who underwent combined resection and ablation, experienced a rebleed at the resection-site that required relaparotomy.

Two events were grade 3: one patient, in whom both CRLM were treated with > 100 W, developed fever requiring rehospitalization and i.v. antibiotics, and another, in whom a single CRLM was treated with > 100 W, experienced biliary tract obstruction along with right portal vein thrombosis.

A total of 4 patients with HCC experienced AEs following 5 procedures (5/44, 11.4%). Three of these AEs were classified as SAEs, accounting for 6.8% (3/44) of the cohort. One grade 5 SAE was reported: the patient developed fever, oliguria caused by acute kidney failure with hyperkalaemia complicated by COVID-19 infection which ultimately led to death of the patient. Two events were grade 3: one acute kidney failure and E. Coli bacteraemia and one contrast induced nephropathy.

Procedure-related details including the power settings, ablation time, approach, number of repositionings and the minimal ablation margins are described in Table 4.

**Table 4:** Procedure-related characteristics for patients with CRLM and HCC

Procedure-related characteristics	CRLM (*N*=168)	HCC (*n*=64)
Duration of ablation in min (median, range)	5.0 (1.0–20.0)	6.0 (3.0–15.0)
N. of repositions (*N*, %)	–	–
None	29 (21.8%)	22 (44.9%)
1	43 (32.3%)	16 (32.7%)
2	31 (23.3%)	9 (18.4%)
3	15 (11.3%)	2 (4.1%)
4	11 (8.3%)	N.R.
≥5	4 (3.0%)	N.R.
Unknown	35	15
Number of tumours treated during the session (median, range)	2 (1–12)	1 (1–5)
Power settings in Watts (per tumour)	–	–
110	2 (1.2%)	N.R.
120	35 (20.8%)	18 (28.1%)
130	36 (21.4%)	9 (14.1%)
140	N.R.	N.R.
150	95 (56.5)	37 (57.8%)
Approach (N, %)	–	–
Percutaneous	77 (93.9%)	44 (100%)
Open	5 (6.1%)	0
Tumour size in mm (median, range)	16 (3.0–50.0)	20.5 (5–51)
Minimal ablation margins	–	–
<1 mm	2 (1.2%)	1 (1.6%)
1–5 mm	15 (8.9%)	7 (10.9%)
>5 mm	151 (89.9%)	56 (87.5%)
Length of hospital stay (median, range)	1 (1–29)	1 (1–11)

Categorical variables are reported as number of patients (%), continuous variables are reported as median (range). N.R. = Not reported

### CRLM: Efficacy and Survival Analysis

**Fig. 1:**
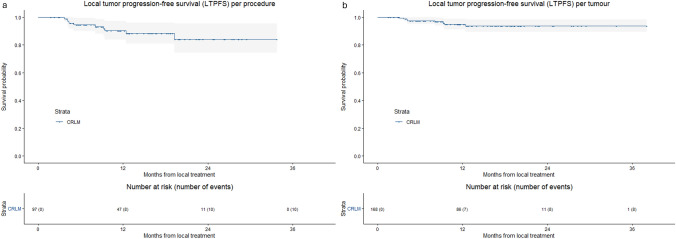
Kaplan–Meier curves with log-rank test of **a** local tumour progression-free survival (LTPFS) per procedure and **b** local tumour progression-free survival (LTPFS) per tumour, from local treatment for CRLM

Technical success was achieved in 97.9% of procedures and 98.8% of CRLMs, with 2 out of 168 CRLMs treated across 97 sessions showing insufficient margins (< 1 mm). The 1-, 2- and 3-year LTPFS per tumour was 94.9%, 93.7% and 93.7% (Fig. 1). LTP was reported in 4.8% (8/168) tumours treated with higher power settings (> 100W), compared to 6.9% (16/233) of CRLM in the historical cohort (*p* = 0.38). All patients with LTP were successfully retreated with MWA; 8 patients had competing risks as they died without developing LTP. No loss of local control was reported (local control 100%), compared to 1 loss of local control in the historical cohort (99.0%; *p* = 0.37).

**Fig. 2:**
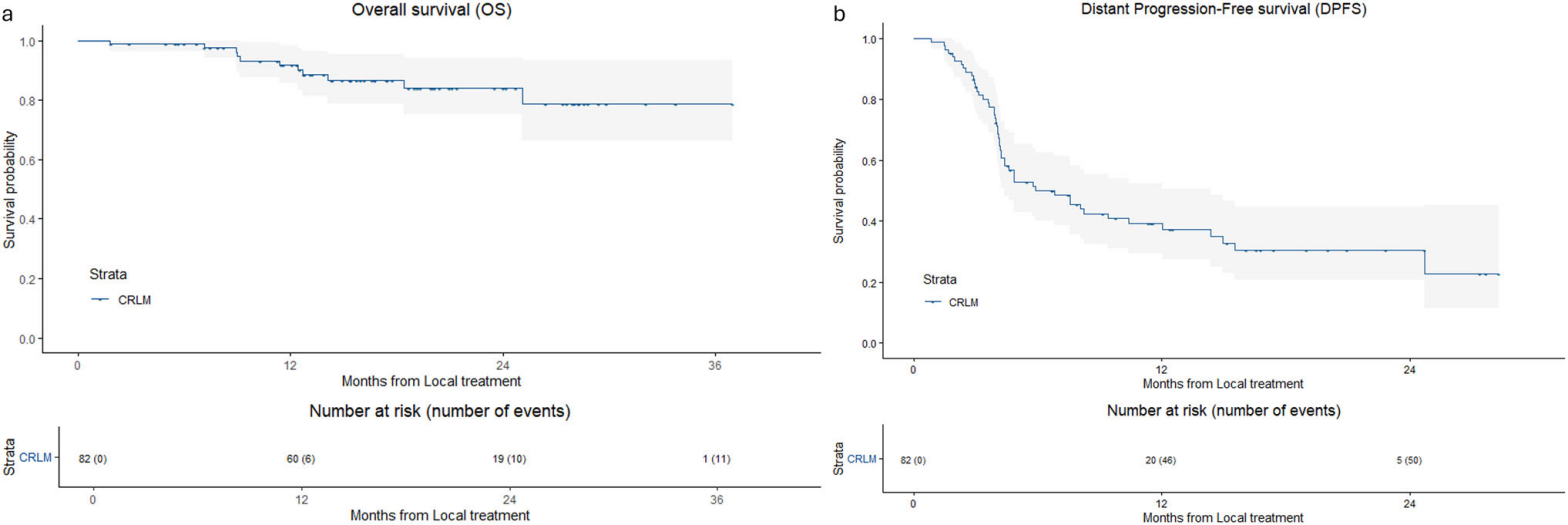
Kaplan–Meier curves with log-rank test of **a** Overall Survival (OS) and **b** Distant Progression-free Survival (DPFS) from local treatment of CRLM

For CRLM the 1-, 2- and 3-year OS was 91.7%, 84.1% and 78.8%, respectively (Fig. 2). Median OS was not reached.

The median DPFS was 5.4 months (95% CI 2.6–8.2). Distant progression was reported in 62.2% of patients. The 1- and 2-year DTPFS was 36.8%, 28.6% (Fig. 2).

For CRLM the median length-of-hospital stay was 1 day (range 1–29). 87.6% of patients were discharged after 1 day. Details regarding procedure related characteristics are provided in Table 4.

### HCC: Efficacy and Survival Analysis

**Fig. 3:**
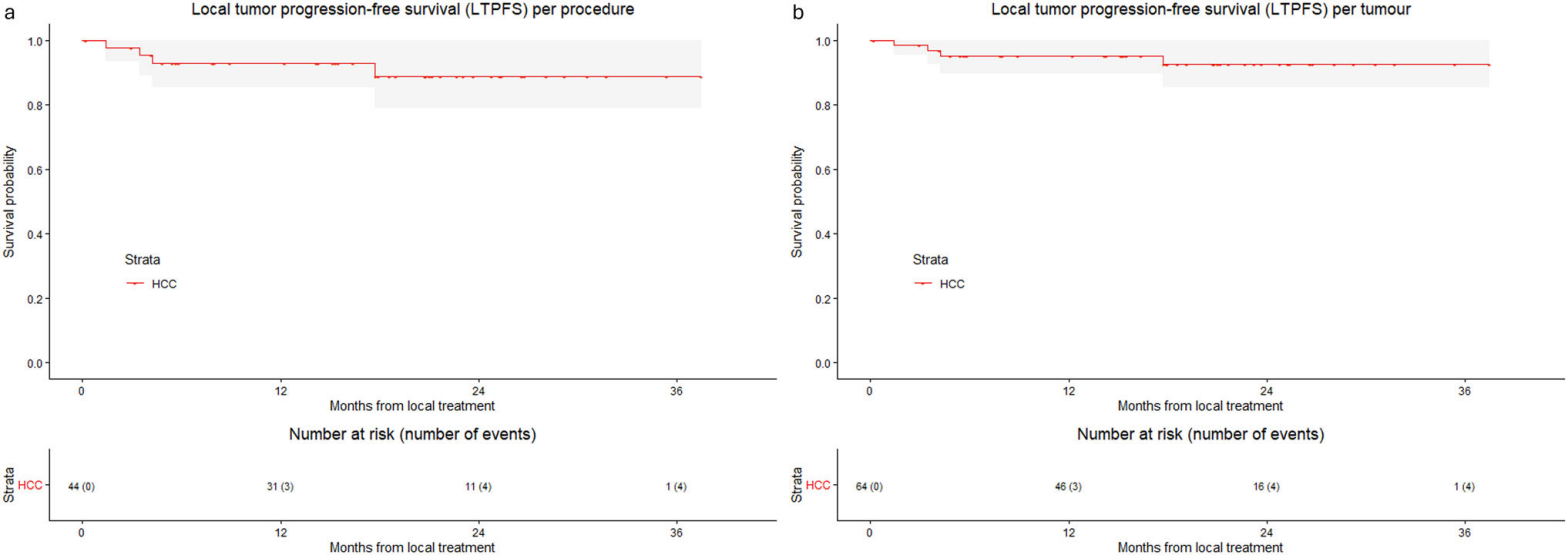
Kaplan–Meier curves with log-rank test of **a** local tumour progression-free survival (LTPFS) per procedure and **b** local tumour progression-free survival (LTPFS) per tumour, from local treatment for HCC

For HCC, technical success was reached in 97.7% of procedures and 98.4% of HCCs, with 1 out of 64 HCCs treated across 44 sessions showing insufficient margins (< 1 mm). The 1-, 2- and 3-year LTPFS per tumour was 95.1%, 92.5% and 92.5%, respectively (Fig. 3). LTP was reported in 6.3% (4/64) of tumours. No cases of loss of local control were reported (local control 100%).

For HCC the 1-, 2- and 3-year OS was 82.7%, 63.5% and 54.4%, respectively. The median OS was not reached. Extrahepatic progression was reported in 9.8% of patients. The 1-, 2- and 3-year DTPFS was 91.8%, 88.8% and 88.8%.

**Fig. 4:**
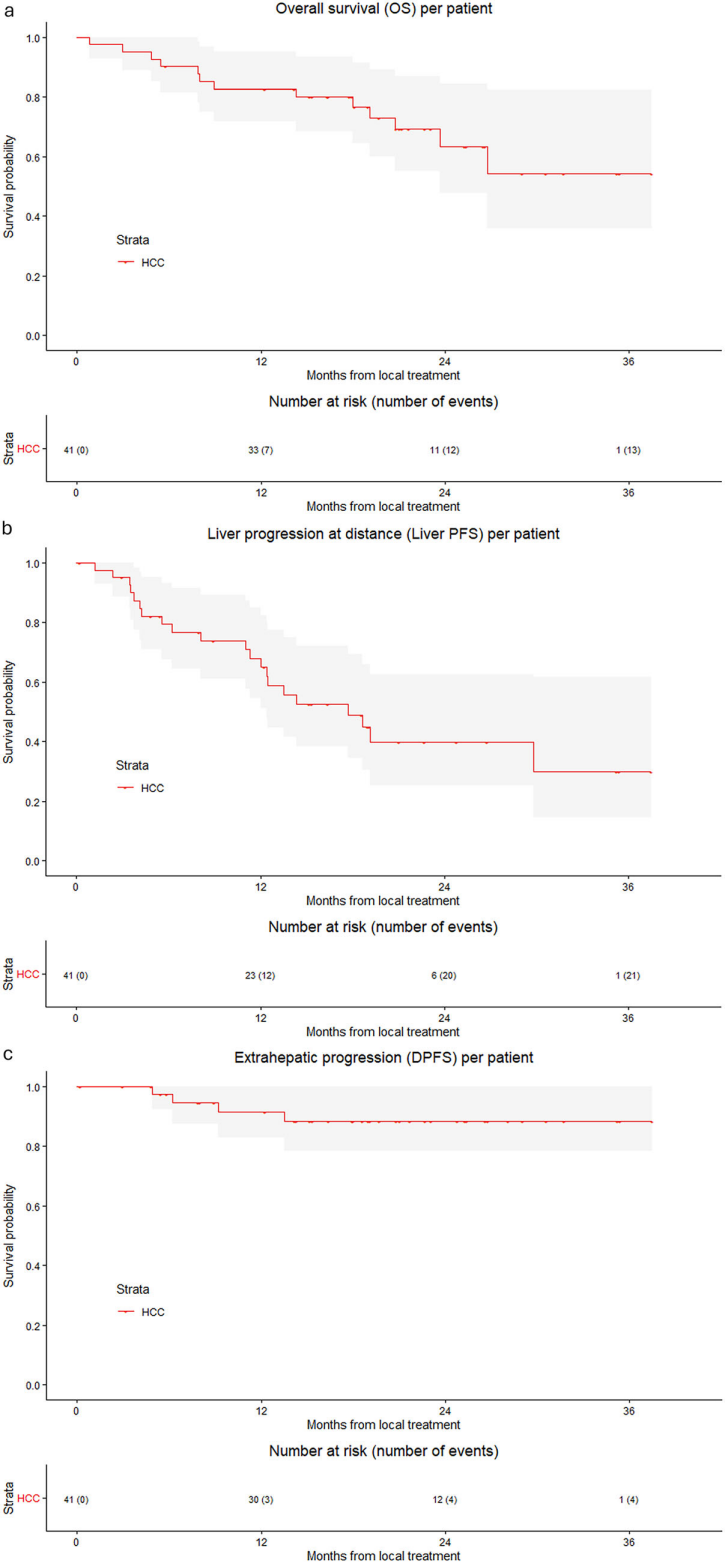
Kaplan–Meier curves with log-rank test of **a** Overall Survival (OS), **b** Liver Progression-free Survival (LPFS) and **c** Extrahepatic progression-free Survival (DPFS) from local treatment of HCC

New HCC at distance from the treated tumours was reported in 21 patients (51.2%). The median hepatic-PFS was 17.7 months (95% CI 9.65–25.7). The 1-, 2- and 3-year hepatic-PFS was 67.9%, 39.8% and 29.9% (Fig. 4).

For HCC the median length-of-hospital stay was 1 day (range 1–11). A total of 88.6% of patients was discharged after 1 day. Procedure related details are provided in Table 4.

### Sphericity Index (SI) and Number of Probe Repositionings

**Fig. 5:**
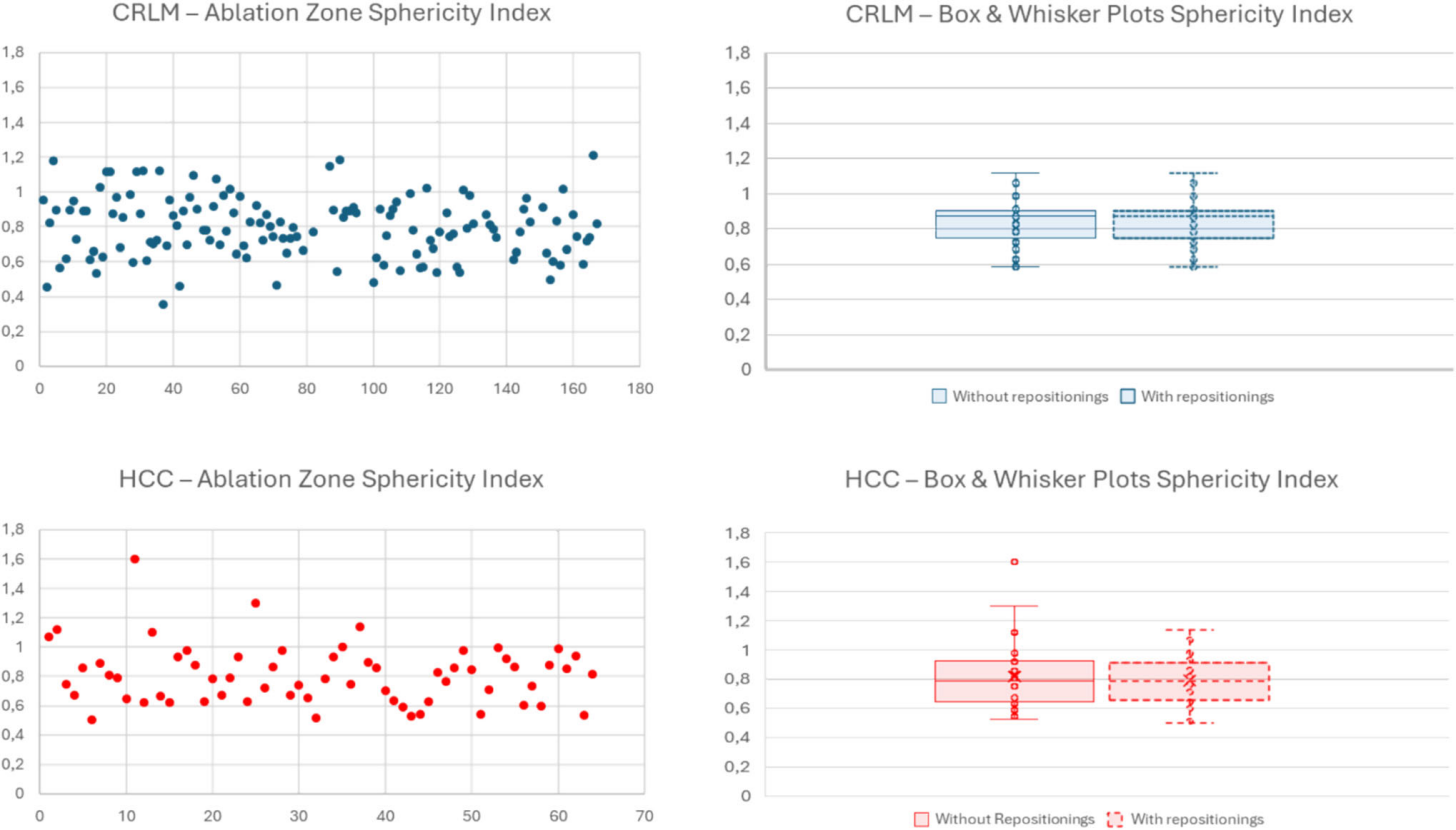
Sphericity index (SI) of the ablation zone with the EMPRINT™ HP. Box and Whisker plots compared sphericity indexes between cases with and without probe repositionings

Median SI was 0.79 (range 0.36–1.21) for CRLM. For the ablations without repositionings the SI was 0.87 (range 0.58–1.12). Median SI was 0.79 (range 0.50–1.14) for HCC. For the ablations without repositionings the SI was 0.79 (range 0.53–1.60). Sphericity indices are shown in Fig. 5. For CRLM and HCC the probe was repositioned at least once in 78.2% and 55.1% of tumours respectively.

## Discussion

The results of this post-market clinical follow-up study confirm the safety and efficacy of Thermosphere™ technology at higher power settings (> 100 W) during MWA in patients with CRLM and HCC. Compared to a historical cohort of CRLM patients treated with conventional power MWA (< 100 W), the safety profile—measured by (serious) adverse events—demonstrated non-inferiority. These results suggest that the increased energy, intended to achieve larger ablation zones and thereby reduce the need for probe repositioning, does not introduce significant additional risk. Furthermore, oncological outcomes, specifically LTPFS, were superior in the high-energy group, with excellent local control achieved for both CRLM and HCC, and no instances of loss of local control observed during the study’s follow-up period. This improved LTPFS may be attributed to the creation of larger, more consistent ablation zones facilitated by higher energy, potentially leading to more complete tumour eradication and a reduced risk of local tumour progression. The absence of locally uncontrolled liver tumours further supports the efficacy of this approach in achieving complete ablation.

The safety and oncological outcomes reported in this series of percutaneously treated patients compare well to previously published literature. In a small series with limited follow-up, Berber and colleagues reported that their early experience with the 150 W device resulted in reduced ablation times and larger ablation zones using a single applicator, compared to their prior use of a 100 W system. The ability to achieve larger ablation zones with a single insertion also enhanced operator confidence, particularly when treating larger tumours [26]. Subsequently, the same research group presented an abstract detailing a study involving 56 patients with 147 liver malignancies treated with a 100 W microwave ablation (MWA) system and another 56 patients with 130 liver malignancies treated using a 150 W system [27]. The clinical and demographic characteristics were similar between the two cohorts. With median follow-up periods of 16 months for the 100 W group and 27 months for the 150 W group, no significant differences were observed in hospital stay (1.6 vs. 1.1 days, *p* = 0.135), 90-day morbidity (4% vs. 7%, *p* = 0.438), or local tumour recurrence (4% vs. 2%, *p* = 0.401). However, when matched for ablation duration, the ablation zones observed two weeks post-procedure were significantly larger with the 150W system compared to the 100W system (*p* < 0.001). Moreover, comparable ablation zones were achieved in a shorter procedure time using the 150W system (*p* < 0.001). Lanza et al. reported 100% technical success with no complications and a primary technique efficacy of 90.2% (*n* = 10/11 patients) when applying higher power settings (> 100 W) during liver tumour ablation [28]. Similarly, Winkelmann et al. reported one minor complication, 100% technique efficacy and no local recurrences during follow-up in twenty-one consecutive patients (22 procedures) treated with MR-guided MWA using an MR-compatible microwave applicator for 28 tumours (9 HCC, 19 metastases) with a mean tumour diameter of 14.6 ± 5.4 mm (range: 6–24 mm) [29].

The use of higher power settings in our study resulted in a median sphericity index of 0.87 (range 0.58–1.12) for CRLM and 0.79 (range 0.53–1.60) for HCC, indicating a geometrically spherical and predictable ablation zone. These values compare favourably with previously reported outcomes achieved using conventional power MWA, suggesting that increased energy delivery does not compromise, and may enhance, the geometric precision of ablation zones. In an ex vivo study, Howk et al. demonstrated the formation of predictable and spherical ablation zones in both hepatic and pulmonary tissue in domestic swine using the Thermosphere™ technology at conventional power settings [30]. Ablations were performed across a range of power levels (45, 75, and 100 W) and durations (1–10 min), with the most consistent and spherical ablation zones observed at 100 W. Consistently, an in vivo study using conventional power settings confirmed the technical success of the Thermosphere™ technology, achieving reliable and spherical ablation zones with short ablation times in a cohort of patients treated for both HCC and metastatic liver tumours [17]. Furthermore, Hendriks et al. compared Thermosphere™ technology with another commercially available system (HS-Amica-Gen 1.0 generator), both operating at a frequency of 2.45 GHz and using conventional power settings (100 W). Their findings indicated that while Thermosphere™ ablations were slightly smaller in volume, they were more spherical and exhibited less variability [23].

The EMPRINT™ HP system employs the same energy modulation strategies as the conventional EMPRINT™ system to achieve consistent, large, and spherical ablation zones. This is accomplished through three primary control mechanisms: thermal control, field control, and wavelength control. Thermal control is achieved by circulating sterile saline through the probe and its coaxial cables, which serves to mitigate the risk of unintended thermal injury to adjacent tissues. Field control is facilitated by the probe’s structural design, which directs electron flow to generate a stable and uniform electromagnetic field, even in the presence of heterogeneous tissue characteristics. Wavelength control is maintained by preserving a consistent electromagnetic wavelength throughout the procedure. By stabilizing the dielectric environment around the probe shaft, a result of continuous saline circulation, which minimizes local variability induced by tissue heating [16]. The current findings suggest that the limitation of smaller ablation zones, associated with conventional EMPRINT™ generator use, appears to have been addressed in the EMPRINT™ HP system through the application of higher power settings, as reflected in the improved LTPFS and excellent LC rates. A higher energy setting may generally be used safely in non-critical locations for a wide range of lesions, achieving comparable ablation zone sizes in shorter durations. However, it appears to offer particular advantages when treating larger lesions or non-spherical, broad-based lesions oriented perpendicular to the probe. Nevertheless, the use of higher power settings may introduce potential challenges that could theoretically impact clinical outcomes. Specifically, increased power settings could add the hypothetical risk of unintended thermal diffusion, potentially affecting critical structures adjacent to the target tumour, despite the implemented saline cooling mechanisms. Furthermore, higher power settings might theoretically exacerbate tissue charring or desiccation, which could disrupt the homogeneity of the electromagnetic field and reduce procedural predictability. A potential for greater impedance mismatch in heterogeneous tissues also exists, which could compromise the efficiency of energy delivery. Consequently, while the application of higher power appears to enhance ablation zone dimensions and efficacy, careful power modulation and monitoring remain crucial to avoid potential complications and ensure consistent outcomes.

This study is subject to several limitations. The retrospective design introduces the potential for selection bias and complicates the adjustment for confounding factors that may have influenced the outcomes. While a historical cohort was used for comparison, the non-randomized nature of the study limits the representativeness of the study cohort to the broader patient population. Furthermore, this study focuses exclusively on a single high-power system and does not include comparisons with other high-power systems. Additionally, the study was conducted at a tertiary referral centre for tumour ablation, and therefore the observed findings may not be equally applicable to centres with less experience in MWA or with different patient demographics. Although the wattage level was determined at the operator’s discretion based on the condition of healthy liver parenchyma, this study did not differentiate between cirrhotic and non-cirrhotic livers in the analysis of adverse events.

In conclusion, this study demonstrates that the use of higher power (> 100 W) MWA in the treatment of CRLM and HCC is safe and effective. Excellent local control can be achieved with higher power settings, without compromising predictability and ablation-zone sphericity, regardless of target location, tissue type or changes in tissue characteristics during the ablation.
